# Gamma-Glutamyl Transferase as a Diagnostic Marker of Metabolic Syndrome

**DOI:** 10.7759/cureus.41060

**Published:** 2023-06-27

**Authors:** Bobbili Tarun Kesava Naidu, Kakarlapudi Santosh Raju, Janapareddi V BhaskaraRao, Nallapati Sunil Kumar

**Affiliations:** 1 General Medicine, Maharajah's Institute of Medical Sciences (MIMS), Vizianagaram, IND; 2 Neurology, Gitam Institute of Medical Sciences and Research, Visakhapatnam, IND; 3 General Medicine, Sri Lalithambigai Medical College and Hospital, Chennai, IND

**Keywords:** diagnostic marker, lft, metabolic syndrome (mets), gamma glutamyl transferase (ggt), ggt

## Abstract

Introduction

The metabolic syndrome consists of metabolic abnormalities that increase the risk of cardiovascular disease (CVD) and cerebrovascular disease. Metabolic syndrome (MetS) is a growing problem worldwide, and substantial efforts have been made in the last years to identify early, minimally invasive blood-based biomarkers for its diagnosis. This study attempted to assess how serum Gamma-Glutamyl Transferase (GGT) performed as an ideal endogenous substance for the diagnosis of metabolic syndrome and hence estimate cardiovascular risks.

Methodology

This study has been undertaken to assess the role of GGT as a marker in the diagnosis of metabolic syndrome and to assess the sensitivity and specificity of GGT in the diagnosis of metabolic syndrome. One hundred and eighty subjects were recruited comprising 90 cases of MetS and an equal number of age and gender-matched controls. Patients were recruited into the study group after satisfying the International Diabetes Federation (IDF) criteria for MetS. GGT values were obtained for both groups apart from other parameters. The patients in the study were also evaluated for the presence of cardiovascular diseases and cerebrovascular accidents (CVA).

Results

Sixty cases have GGT levels above the normal range (55 in males and 38 in females), and none in the control group have GGT levels above normal. This difference is statistically significant (*p*=0.01). The sensitivity was found to be 67% and 94% for males and females respectively. The specificity was found to be 100% and 98% for males and females respectively. Among the 90 cases, 20 (22.2%) patients developed cardiovascular disease. None in the control group developed cardiovascular disease. This difference is statistically significant (*p*<0.05).

Conclusion

Serum GGT appears to be an easily available and fairly good marker for diagnosing patients with metabolic syndrome and is independent of other parameters of metabolic syndrome. It is also a strong predictor of cardiovascular disease. Hence GGT can be considered a potential marker for the evaluation of patients with metabolic syndrome.

## Introduction

Metabolic syndrome (MetS) is considered a group of metabolic abnormalities that consist of hypertension, atherogenic dyslipidemia, obesity, and resistance to insulin that increase the risk of getting affected with cardiovascular and/or cerebrovascular diseases [1]. The increase in the prevalence of central obesity within the global population makes MetS a relevant concern among medical professionals. The general population in India is also facing a threat of increased risk of atherosclerotic cardiovascular diseases (ASCVD) due to the high prevalence of MetS among them. Evidence reports a global prevalence of MetS to be 20-25% [2]. It is approximately 31.4% of the Indian population [3]. World Health Organization (WHO) has reported an increased prevalence of 46.4% in the southern states of India, whereas evidence has also reported a prevalence rate between 18.3 and 41% [4]. Amidst all complications, cardiovascular incidents are found to be causing significant events of morbidity and mortality.

Early recognition of metabolic syndrome helps in timely intervention through lifestyle changes and pharmacotherapy to reduce morbidity and mortality arising from cardiovascular issues. Several markers have been identified such as adiponectin in order to quantify the increased adipose tissue. But their relative use is questionable as they are not proven cost-effective. Serum gamma-glutamyl transferase (GGT) was proposed as a predictive biomarker as GGT levels correlate with the rising risk of type 2 diabetes as well as MetS. The Framingham Heart Study exemplified that GGT is directly associated with body mass index (BMI), blood pressure, triglycerides, glucose levels in the blood as well as LDL (low-density lipoprotein) cholesterol. The risk of MetS rises with higher levels of GGT. Many extensive population studies of the Western world confirmed the relationship between GGT and MetS [5]. There is a paucity of evidence of their association in the context of the Indian population. Currently, the emphasis is to identify a feasible and less-expensive marker in order to identify and promptly predict the incidence of the syndrome at an early stage in the Indian population. GGT is considered to be one of the feasible biochemical markers, and is considered to be easily accessible and relatively inexpensive, and is also routinely evaluated as a component of liver function tests.

## Materials and methods

This study is being done to analyze the effectiveness of GGT as a biochemical marker to diagnose metabolic syndrome (MetS) and to evaluate the specificity and sensitivity of GGT as a biochemical marker in the diagnosis of MetS. In this prospective observational study, the patients who reached out to the services of the outpatient department, as well as those who were admitted as inpatients at Maharajahs Institute of Medical Sciences (MIMS), Nellimarla, Vizianagaram from January 2020 to June 2021, were included in the study. Among 90 cases, patients above the age of 18 years, patients having Central obesity (measured waist circumference of ≥90cm for men, ≥80cm for women), and patients with any two of the following four factors: patients with raised triglyceride (TG) levels of ≥150mg/dl or those who underwent treatment for the same, patients with an increased systolic blood pressure (BP) of ≥130mmHg and diastolic BP of ≥85mmHg, or those with a previous history of hypertension, patients with a decreased high-density lipoprotein (HDL) cholesterol of <40mg/dl or those who underwent treatment for the same and patients with an increased fasting plasma glucose of ≥100mg/dl or with a previous history of diabetes were included in the study. Ninety age and sex-matched healthy controls were included in the study. Patients who were diagnosed with liver diseases, both acute and chronic, hypothyroidism, malignancy, severe renal insufficiency, alcohol consumption, and those who were under regular medications such as oral contraceptives, antiepileptics, trimethoprim, erythromycin, sulphamethoxazole were excluded from the study in both the groups. The institutional ethics committee, Maharajah's Institute of Medical Sciences, Nellimarla, has approved the study (IEC/43/20).

Data was collected using a pretested semi-structured questionnaire, clinical examination, and investigations. The GGT estimates were analyzed for all the participants, both in the experimental and control group. The GGT was estimated according to the guidelines advised by the International Federation of Clinical Chemistry (IFCC). This methodology utilizes the substrate L-gamma-glutamyl-3-carboxy-4--glutamyl transferase to catalyze the transfer of oglutamyl-3-carboxy-4-nitranilide (GCNA) to glycylglycine, which releases 5-amino-2-nitrobenzoate, absorbing at 405-glutamyl transferase activity, which is assessed with the help of a biochromatic (405, 600 nm) rate technique. The normal reference range values for GGT are 55 IU/L and <30 IU/L for males and females, respectively. Data was analysed using descriptive statistics mean and standard deviation for quantitative variables. Frequency and percentages were used for qualitative data. The Chi-square test was performed for comparing qualitative attributes and the student t-test for quantitative attributes, and p<0.05 was considered statistically significant. Data is also described in tables and graphs. The specificity and sensitivity analysis were performed for GGT for the diagnosis of MetS. The results were analyzed using Microsoft Excel 2013.

## Results

Out of the 90 cases, 47 (52.22%) were males, and 43 (47.77%) were females. Among the 90 controls, 42 (46.6%) were males, and 48 (53.4%) were females. The difference in genders in both groups is not statistically significant (*p=*0.5). Among the 90 cases, 47 (52.2%) belonged to the age group 50-59, 31 (34.5%) belonged to the age group 40-49, 10 belonged to the age group 60-69, and one each to the age group 30-39, and greater than 69. The mean age of cases was 51.2±8.2 years and the mean age of controls was 54.2±7.6 years. The difference between the age among both the groups was not statistically significant at (*p=*0.2). Among cases, 67 (74.5%) patients belonged to urban areas, and the remaining belonged to rural areas, and this difference is statistically significant at p<0.05. Among controls, 61 (67.77%) belonged to urban areas. The difference in urban to rural populations among cases and controls was not statistically significant (p=0.08). Among cases, 52 (57.8%) patients have BMI between 25 and 30, and 36 (40%) patients have BMI greater than 30.74 (82.2%) people in the control group have BMI less than 25 and none more than 30. This difference is statistically significant at a *p*-value of 0.01. A comparison of GGT values between cases and controls is shown in Table 1.

**Table 1:** GGT values in cases and controls GGT: Gamma-glutamyl transferase

Variable	Subcategory	Cases	Controls	P-value
GGT (IU/L)	<55/38	30 (36.6%)	90 (100%)	0.01
	>55/38	60 (63.4%)	0 (0%)	

60 out of 90 cases have GGT levels above the normal range (55 in males and 30 in females), and none in the control group have GGT levels above normal. This difference is statistically significant as p value is 0.01. Comparison of liver function enzymes is given in Table 2. 

**Table 2:** Liver function enzymes in cases and controls AST: Aspartate aminotransferase; ALT: Alanine aminotransferase; ALP: Alkaline phosphatase

Variable	Subcategory	Cases	Controls	p-value
AST (IU/L)	Less than or equal to 40	32 (35.5%)	90 (100%)	0.01
	Greater than 40	58 (64.5%)	0	
ALT (IU/L)	Less than or equal to 40	53 (58.8%)	90 (100%)	0.01
	Greater than 40	37 (41.2%)	0	
ALP (IU/L)	Less than or equal to 150	44 (48.8%)	90 (100%)	0.01
	Greater than 150	46 (51.2%)	0	

Comparison of fatty liver (in Ultrasound) among cases and controls is shown in Table 3.

**Table 3:** Fatty liver changes among cases and controls USG: Ultrasonography

USG abdomen (Fatty liver)	Cases	Controls	P-value
No	23 (25.5%)	69 (76.7%)	0.01
Yes	67 (74.5%)	21 (23.3%)	

Of 90 cases 20 patients developed cardiovascular disease. None in control group developed cardiovascular disease. This association is statistically significant at p-value 0.01. Of 90 cases, 44 patients developed cerebrovascular disease. Four in the control group developed cerebrovascular disease. This difference is statistically significant at p-value 0.01. Presence of absence of elevated GGT in metabolic syndrome is shown in Table 4.

**Table 4:** Table showing elevated GGT among cases and controls GGT: Gamma-glutamyl transferase

GGT	Subjects with metabolic syndrome	Subjects without metabolic syndrome	Total
Elevated	60	0	60
Normal	30	90	120
Total	90	90	180

For GGT as a diagnostic marker in metabolic syndrome, Sensitivity is 66.6%, specificity is 100%, positive predictive value is 100% and negative predictive value is 75%.

## Discussion

Gamma-glutamyl transferase (GGT) has long been thought to be an indicator of both alcohol abuse and hepatobiliary dysfunction. It is an enzyme that catalyzes the initial stage of glutathione's (GSH) extracellular breakdown and may contribute to atherogenesis. In addition to alcohol, obesity has been discovered to have a significant impact on blood GGT. Furthermore, mounting evidence indicates that elevated serum GGT levels are associated with various metabolic disorders, including glycemic disorder, hypertension, hypertriglyceridemia, and low HDL (high-density lipoprotein) cholesterol. Recent epidemiological studies have demonstrated that GGT is involved in a number of common pathophysiological processes, such as oxidative stress and lipid peroxidation, which are crucial to the pathophysiology and development of insulin resistance and the MetS. Additionally, when GGT and other hepatic markers were evaluated, GGT was the primary indicator of type 2 diabetes [6-9]. Low-density lipoprotein is probably being oxidized inside the plaque by a GSH/GGT-dependent iron reduction mechanism. Oxidative stress is a likely mediator in this situation. Recent discoveries on the role of GGT's pathophysiological backdrop in the development and progression of atherosclerosis seem to be supported by pertinent epidemiological data as a predictor of cardiovascular risk [8,10]. There is a paucity of literature in the Indian context assessing the influence of MetS in the adult population and the need for early identification and initiation of preventive measures. This study also identified the effectiveness and role of GGT as a marker in identifying the MetS and its utility in predicting the risk for CVDs.

The study recruited 180 subjects, comprising 90 patients diagnosed with MetS and 90 age and gender-matched controls. In this study, MetS was most common in the fifth decade of life; 52.2% of cases were seen in this age group. The mean age of the patients in the experimental group who were diagnosed with MetS was 51.2±8.2 years whereas the mean age of the control group was 54.2±7.6 years. By t-test, the p-value was 0.2, which is statistically insignificant. There were 63.3% males and 36.7% females in the study group, whereas the control group consisted of 46.6% and 53.4% males and females respectively. The difference was statistically significant with a p-value of 0.02 for the Chi-square. The study by Kasapgolu et al. [4] found the mean age of the patients diagnosed with MetS was 51.3±3.2 years with 62% of females and 38% of males in the recruited population. Our study contradicts this as the proportion of males were higher in this study. The gender distribution of our study and Kasapgolu et al. [4] was not similar as MetS is more prevalent in males (63.3%) in our study. In the study conducted by Ramli et al., the risk of MetS, irrespective of criteria, is higher in the elderly and was found to be significantly higher in those aged above 60 years [11]. In our study, 67 (74.5%) patients are from urban areas, while 23 (24.5%) are from rural areas. The results would have been influenced by the study center and the population in which the study was conducted, as the major source of recruited patients was from a tertiary care center, which falls in an urban setup. But the results are consistent with other evidence reported in patients with MetS [5]. The study found a mean waist circumference of 96.9±3.4cm in the MetS group, whereas the subjects in the control group had a mean waist circumference of 77.3±1.7cm. The mean BMI for the patients in the MetS group was 29.7±2.9, and it was 24.21±1.2 for the subjects in the control group. The study calculated a mean waist-hip ratio of 1.10±0.08 for the patients in the MetS group, whereas the control group subjects demonstrated a waist-hip ratio of 0.73±0.51. The study was consistent with the articles available in the literature [12]. In our study, 53 (58.9%) of the patients in the MetS group had systolic BP of more than 130 mmHg. When compared with the control group, the difference by Chi-square is statistically not significant as the p-value is 0.17. The study done by Kasapgolu et al. [4] also reported consistent results with systolic BP of 137.1±10.6mmHg and diastolic BP of 86.7±7.2 mmHg in the recruited population. Interestingly, 74 patients (82.3%) of the MetS group had diastolic BP >80mm of Hg, and the finding is statistically significant (p-value=0.01) for Chi-square. However, the influence of elevated DBP on MetS is still questionable and must be subjected to further qualitative studies. The study also observed an association between central obesity in the development of metabolic syndrome.

Evidence suggests the presence of central obesity and hypertension as the major indices influencing the development of MetS regardless of the criteria recommended for diagnosing MetS [3]. Literature has identified the presence of central obesity in 80% of the study population with MetS, suggesting its role as the major influence in the mechanism causing MetS [13]. The mean GGT levels in the MetS group was 52.44±6.01 IU/L whereas the subjects in the control group had a mean GGT value of 34.57±8.20 IU/L. The MetS group had a statistically significant greater level of GGT than the control group (*p-*value=0.01). The study by Kasapgolu et al. [4] demonstrated the mean GGT in the study and control group to be 40.9±10.2 and 21.0±7.1 respectively. The validity of the study was computed with a GGT reference value of ≥ 55 IU/L and ≥ 38 IU/L, for males and females respectively. The literature reported the sensitivity and specificity of GGT to be 66.6% and 100% in identifying and diagnosing MetS in patients. Our study observed that although a majority of the subjects had a GGT value below the normal ranges, they are clustered at the upper range of normal. Among the 84 (93%) patients, 22 (25%) fall in the upper range limit of the normal values. We suggest the need for considering those who represent the upper range of the normal limits to be helpful in predicting the MetS. The study by Rantala et al. [14] demonstrated the association between GGT and MetS. They reported a statistically significant relationship between the components of MetS with GGT after adjusting for age, BMI, and alcohol intake. The study by Sakugawa et al. [15] also could demonstrate the relationship between GGT levels and components of MetS. Of the 53 patients with SBP >130mmHg, 36 (60%) had GGT levels above the reference range. Seventeen cases had GGT within the reference range, and this difference is statistically not significant. Out of 59 cases with FPG (fasting plasma glucose) >100 mg/dL, 38 had higher GGT levels. Twenty-one cases had GGT levels within the normal range, and this difference is statistically not significant. Out of the 56 subjects with lower HDL levels (<40 IU/L and < 50 IU/L for males and females respectively), 35 were found to have GGT levels above the normal ranges, whereas 21 had within the reference range. Among the patients identified as having hypertriglyceridemia (68), 44 (49.5%) had increased levels of GGT. The results support the findings that GGT has a direct correlation with hypertriglyceridemia. Higher levels of GGT were found to directly correlate with the prevalence of CVD in patients with MetS according to our study (*p*=0.002, 95%CI). Ruttman et al. [16], demonstrated an independent association of GGT with death of cardiovascular origin. Out of 44 patients with stroke (ischemic and hemorrhagic) in the study group, 38 cases have elevated GGT levels and only six have GGT in the normal range. This difference is statistically highly significant by Chi-square with a p-value of 0.0001. In a study by Ruttman et al., serum GGT levels were significantly associated with stroke in males, both hemorrhagic and ischaemic, but not in females [16].

The MetS group had a mean aspartate aminotransferase (AST) of 33.83±07 IU/L and alanine aminotransferase (ALT) of 38±13.74 IU/L, whereas the control had 24.8±3.96 IU/L and 25.4±4.41 IU/L, respectively. The results illustrate increased levels of hepatic transaminases in subjects identified as having MetS, although the values were within the normal range. The results of the study conducted by Kasapgolu et al. [4] concluded the presence of normal levels of liver enzymes coexisting with the patients identified to have MetS. This is because of the findings in which 91.2% of the subjects with MetS had transaminases within the normal range, and 83.4% of the subjects had normal GGT levels. They pointed out elevated levels of the parameters when compared to that of the controls even though the values are within the normal range [4]. The abnormality in the liver function tests of patients with diabetes is mostly related to an increased level of GGT. GGT estimation has been found to be an independent predictor for the incidence of type 2 diabetes in both males and females. The study by Rantala et al. [14] also demonstrated the significant association between GGT and MetS, as well as the indices of MetS. The results of the study by Rantala et al. were adjusted for age, BMI, and the quantity of alcohol consumed [14]. The results of our study identified elevated levels of GGT, either above the normal levels or at the upper limit of normal, in the subjects diagnosed with MetS. The study also identified elevated GGT levels in all patients with a history of cardiovascular diseases (CVD) than those without. The levels of GGT were significantly correlated with hypertriglyceridemia. GGT was found to be 67% sensitive in males and 94% in females in diagnosing MetS, whereas the specificity levels in males and females were 100% and 98%, respectively [14].

Limitations

The sample size was small, and other confounding factors that might have affected the outcomes were not studied. Large multi-centric trials are to be conducted to further strengthen the correlation.

## Conclusions

GGT values were significantly higher in patients with metabolic syndrome. Cerebrovascular accidents and cardiovascular diseases were more significant in the patients with MetS. Since the assessment of GGT is easily available and performed as part of liver function tests, it can be considered a reliable marker for metabolic syndrome and to predict the risk of cardiovascular and cerebrovascular diseases.
